# Growth of the hard palate in infants with Down syndrome compared with healthy infants—A retrospective case control study

**DOI:** 10.1371/journal.pone.0182728

**Published:** 2017-08-10

**Authors:** Daniel Klingel, Ariane Hohoff, Robert Kwiecien, Dirk Wiechmann, Thomas Stamm

**Affiliations:** 1 Dental Office Dres. Pape, Rheda-Wiedenbrück, Germany; 2 Department of Orthodontics, University of Münster, Münster, Germany; 3 Institute of Biostatistics and Clinical Research, University of Münster, Münster, Germany; 4 Department of Orthodontics, Medizinische Hochschule Hannover, Hannover, Germany; Medical University of South Carolina, UNITED STATES

## Abstract

**Objective:**

To investigate morphological differences of the hard palate in infants with Down syndrome (DS) compared with a volumetric-matched control group (CG).

**Methods:**

Trial design: retrospective case control study. Based on inclusion and exclusion criteria, plaster casts of edentulous maxillae of 40 DS infants (20 females and 20 males, aged 221.3 ± 132.4 days) and 40 CG infants (20 females and 20 males, aged 53.9 ± 87.2 days) were digitized and converted into 3-dimensional stereolithography data. An automated landmark- and investigator-independent method for assessing two-dimensional measurements such as width, depth, and length of palate, as well as palatal index and the 3-dimensional volume, were used.

**Results:**

Matching DS and healthy CG infants by age, we found reduced sizes in all linear and volumetric measurements in the DS group. Matching both groups by palatal volume, we found no differences between the groups according to palatal width (p = .93), palatal depth (p = .32), and palatal index (p = .31). Control infants with the same palatal volume compared with the DS infants were about 151 days younger, 95%-CI = [102, 200] (Hodges-Lehmann estimator). Except for palatal length and palatal volume, the growth pattern of DS palates decreased irregularly at age 6 to 9 months.

**Conclusions:**

The palate of DS infants in the first 6 to 9 month of life is of normal shape but considerably smaller compared with healthy normals. From 6 to 9 months onward, the growth pattern of the hard palate in DS infants decreases irregularly. High-arch-constricted palates could, therefore, be interpreted as secondarily acquired in later life. We therefore speculate that it could be advantageous to begin oral muscular stimulating therapy between 6 and 9 months of age which may prevent palatal shape alterations and enhance oral function which also contributes to maxillary development.

## Introduction

Various cranio- and orofacial conditions have been described in patients with Down syndrome (DS). Despite nuchal thickness, maxillary hypoplasia may play an important role in prenatal ultrasonographic diagnostics [1, 2] because trisomy 21 fetuses have significantly shorter maxillary lengths than do normal fetuses [1]. Pre- and postnatal growth disturbances of the maxillonasal complex lead to the appearance of an underdeveloped midface and a prognathic mandible in later life [3–5].

There is disagreement which orofacial features are caused by the genetic disorder and which are secondarily acquired in later life. Knowing which features are epigenetic [6] is essential for specific treatment of individuals with Down syndrome. Craniofacial hard tissues in adolescent and adult DS patients are well studied. The main findings are a small cranial base, large cranial base angle, reduced maxillary length, and increased mandibular length [7–9], causing skeletal Class III malocclusion. Concerning Moss’s functional matrix theory [10], development of the alveolar process is due to the inductive growth potential of the teeth. In view of this, delayed tooth eruption and hypodontia in DS individuals [5, 11] contribute to the severity of malocclusion.

Not only the jaw position but also morphological distortion can impair orofacial function. The hard palate of DS adolescents is often described as high arched and constricted or narrow [4, 11, 12], but data in the literature are contradictory [13, 14], depending on patient age [Fischer-Brandis, 1985] of the studied DS group. A narrow maxilla, meaning a growth disturbance in the transverse dimension, cannot be explained by the general growth retardation that leads to small dimensions in all planes of space. Therefore, soft tissue disturbances like hypotonia of the tongue and the perioral and masticatory muscles [4, 15] and a large, protruding tongue [16, 17] are considered to be secondary causes of a high and narrow palatal vault.

Tongue sucking habits or deep tongue pressure at rest would be both leading to a narrowing of the maxilla, the first by enhancing the buccal pressure, leading to compression tones on the alveolar process, the second by generating a missing counter force of the tongue from inside while pressure from the buccal side remains the same.

The majority of studies assessed adolescent or adult DS palates, but measurements on infants are missing. Moreover, only few studies used control groups, and some were matched by sex and age. It can be shown that palatal volume and palatal surface are robust against shape differences and deformities of the palate compared with linear measurements [18, 19]. Therefore, the current study focuses on the infant period, and it is the first study that considers volumetric measurements for matching a control group. The aim of this study is, therefore, to determine whether there is a morphological difference in the hard palate between DS infants and healthy, full-term infants.

## Materials and methods

### Subjects

Edentulous maxillary casts of infants treated with a stimulating plate [20] were retrospectively identified from medical records of the Department of Orthodontics, the University Hospital Münster, Germany. Medical history were reviewed to search for trisomy 21 or Down syndrome.

Inclusion criteria for the DS group were (1) known trisomy 21, (2) plaster cast of the edentulous upper jaw prior to treatment, (3) the whole alveolar process with tubera maxillae and palatal vault perceivable on the plaster cast. Exclusion criteria were (1) other pathological conditions than trisomy 21, especially clefts, trisomy 18, and other syndromes, (2) poor quality of the cast, (3) erupting teeth.

Of 233 selected infants treated with a stimulating plate 57 remained for inclusion in this study. During the matching process, an additional 17 patients were excluded because of lack of comparable cast from the healthy infant group. Finally, 40 DS infants (20 females and 20 males) aged 221.3 ± 132.4 days remained for analysis. A control group of healthy term-born infants (20 females and 20 males, aged 53.9 ± 87.2 days) was established by using digital plaster casts from a clinical trial registered under clinicaltrials.gov (NCT00408746).

Permission to conduct this study was given by the Ethics Committee of the General Medical Council Westfalen-Lippe and the Medical Faculty of the Westphalian University of Münster, Germany. An informal request for using retrospective, anonymised data of DS infants was approved on September 4, 2013, prior to the study. Written informed consent was not obtained on retrospective data. Data from term-born infants (control group) were taken from a clinical trial registered under clinicaltrials.gov (NCT00408746). Prior to this study written informed consent was obtained from parents of term-born infants.

### Matching the control group

Edentulous upper jaw casts of healthy infants are not routinely available to match the DS group by age. We therefore relied on digital casts of a study where healthy term and preterm infant palates were compared [18]. Because of age discrepancies between term and preterm infants, palates were compared by linear and 3-dimensional measurements. It could be shown that the volume of the palate is a valuable parameter to assess palatal growth [18]. We therefore used the same methodology to establish a healthy control group. Term infants were matched by gender and the calculated volume of the palate. Casts with the lowest differences in palatal volume (<50 mm^3^) were assumed as nearly identical concerning 3-dimensional growth.

### Data acquisition and analysis

**Fig 1:**
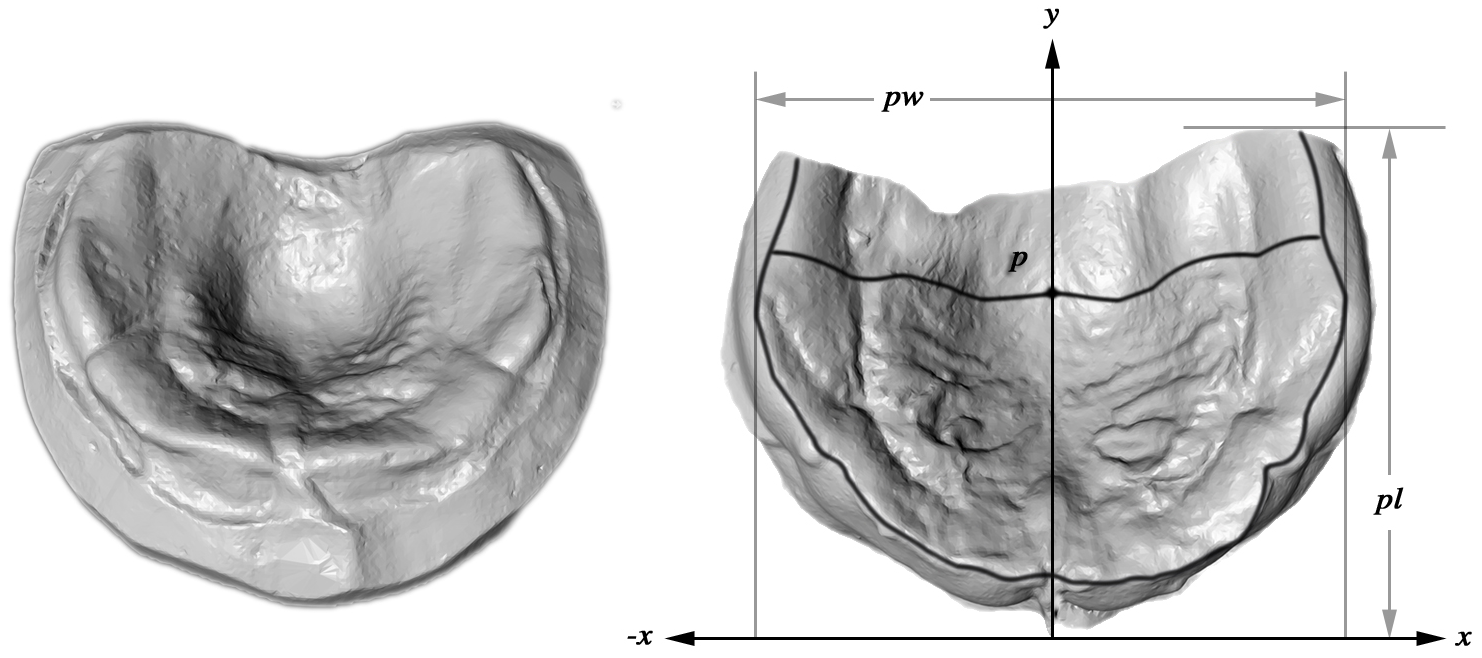
Measurements obtained from digitized plaster casts. Left: Digital plaster cast of a newborn’s palate. Right: Segmented cast. All areas of the cast that were not covered by impression material were digitally removed. The segmented casts were then oriented in a 3D coordinate system according to the raphe palatina mediana and by symmetrical alignment of the alveolar ridge towards a horizontal reference plane. The highest points (z-plane, view direction) of the alveolar bone constitute the alveolar ridge (black line). Point p is the deepest point of the digital cast in the z-plane. A transverse (black) section line passing through point p represents the distal border of the palate for volume calculations. Palatal length (pl) is the longest distance parallel to the y-plane between the most anterior and the most posterior point. Palatal width (pw) is the longest distance parallel to the x-plane and perpendicular to the y-plane between 2 surface points on the right and left side of the alveolar ridge. Palatal depth (pd) is the longest distance parallel to the z-plane between the highest and the deepest point of the cast. The calculated palatal volume is the volume enclosed by the maximum contour line of the alveolar ridge and the dorsal border line determined by the deepest point p.

All plaster casts of the DS group were digitized with the ATOS II system (GOM mbH, Braunschweig, Germany) and were exported as stereolithography (STL) files for further processing. Software was developed to extract linear and 3-dimensional measurements from the STL files. Detailed processing and theoretical background are clearly pointed out by [18]. The reliability of this landmark-and-investigator-independent method has already been proven. The error of this method ranges from 0.56% to 2.66% in “feature dependent, linear distances” and 4.34% in “feature-dependent volume calculations [18]. The measurements in this study comprised width (pw), depth (pd), length (pl), and volume (vl) of the palate (Fig 1), as well the dimensionless palatal index (pi = depth/width). Two further measurements were obtained: the distance in the y-plane from the most anterior point to the point of maximum palatal depth (y-pd) and to the maximum palatal width (y-pw).

### Statistics

Descriptive statistics were performed using the software SPSS (IBM SPSS Statistics 21 for Windows, IBM Corp, Somers, NY). Differences in measurements between the 2 groups were assessed by the Mann-Whitney *U* test, and Hodges-Lehman estimator additionally 95%-confidence intervals (CI). Inferential statistics are intended to be exploratory (hypotheses generating), not confirmatory, and are interpreted accordingly. The comparisonwise type-I error rate is controlled instead of the experimentwise error rate. Local significance level is set at 0.05. No adjustment for multiple testing is performed. Therefore, an overall significance level is not determined and cannot be calculated.

## Results

### Matching

**Fig 2:**
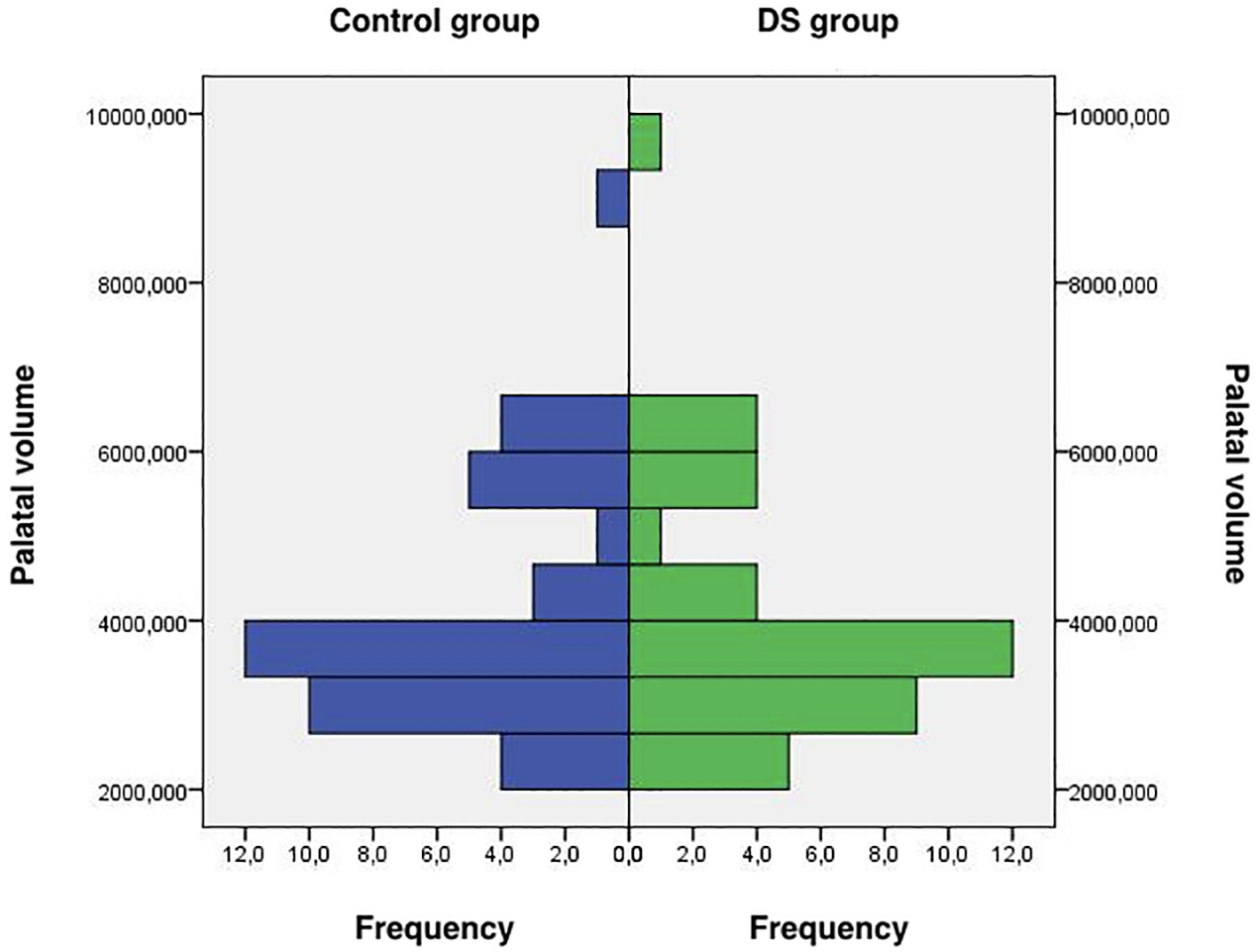
Results of matching DS patients with healthy controls according to the parameter palatal volume [mm^3^].

The DS group was on average 221.3 ± 132.4 days old—an age range in which parents normally seek treatment for proper oral development of their DS-affected children. Plaster casts of healthy infants are not available in this age range; therefore, the groups could not be matched by age. The parameter “palatal volume” was used for the matching process because it represents a 3-dimensional measurement and could be seen as a better representation of growth and development compared with linear measurements. Results of the matching process are presented in Fig 2.

### Measurements

The measurements obtained are presented in Table 1. Except for age (p < .001) there were no differences in the parameters of the hard palate between the 2 groups. The DS group had the same dimensions as the average 151-days-younger control group.

**Table 1:** Age at time of impression taking and measurements obtained from maxillary casts of the DS and control group.

Measure	Hodges–Lehmann estimator / [95%-CI]	p	Control Group*	DS Group*
age (days)	151 / [102, 200]	< .001	53.9 ± 87.2	221.3 ± 132.4
Palatal width (mm)	-.063 / [-1.316, 1.254]	.931	32.3 ± 2.7	31.9 ± 2.8
Palatal depth (mm)	.326 / [-.281, .866]	.317	9.2 ± 1.3	8.9 ± 0.9
Palatal index (depth/width)	.007 / [-.008, .021]	.312	0.2 ± 0.0	0.2 ± 0.0
Palatal volume (mm^3^)	8.470 / [-443.347, 524.845]	.931	4094.4 ± 1456.3	4075.4 ± 1552.8
^1^Y-pd (mm)	-.065 / [-.970, .710]	.814	18.5 ± 1.7	18.6 ± 2.3
^2^Y-pw (mm)	-.890 / [-2.160, .240]	.098	18.9 ± 3.0	19.8 ± 2.6
Palatal length (mm)	1.125 / [-.510, 2.670]	.167	29.5 ± 4.3	28.5 ± 2.4

*Data presented as Mean ± SD.

^1^Distance in the y-plane from the most anterior point to the point of maximum palatal depth.

^2^Distance in the y-plane from the most anterior point to the point of maximum palatal width.

**Fig 3:**
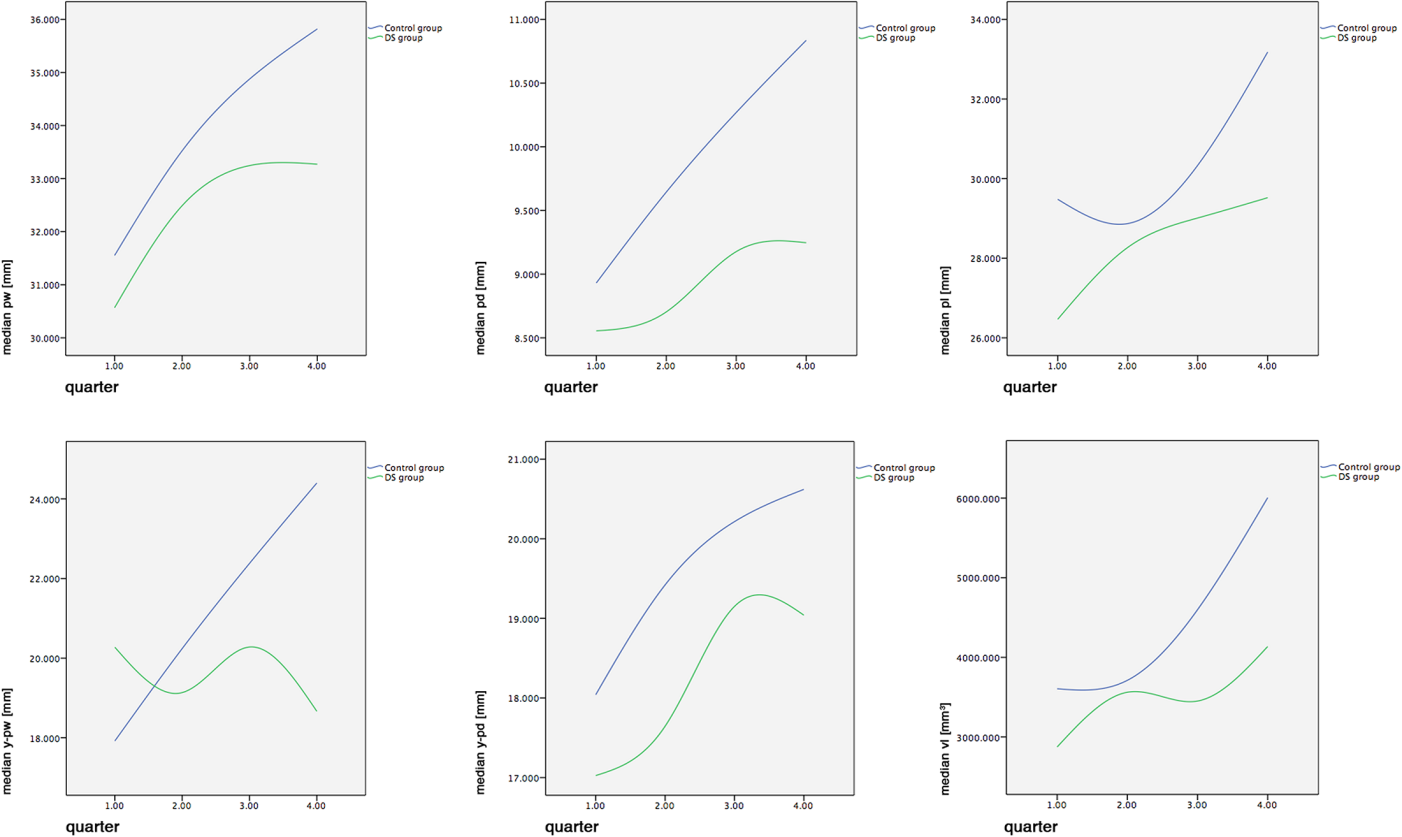
Measurements in relation to age at the time of plaster cast fabrication. Age aggregated into quarters (Q1: ≥1 day <92 days; Q2: ≥92 days <183 days; Q3: ≥183 days <274 days; Q4: ≥274 days <365 days). Represented are the median values per quarter. Q1 (n = 37), Q2 (n = 20), Q3 (n = 7), Q4 (n = 9); Q5 and Q6 are not displayed because of missing controls.

To assess the parameters in relation to age we followed the methodology of Hohoff et al. [18]. Therefore, the data were aggregated into quarters (Q1: ≥1 day <92 days; Q2: ≥92 days <183 days; Q3: ≥183 days <274 days; Q4: ≥274 days <365 days). The age-aggregated medians of the healthy control group showed a characteristic pattern. All measured parameters increased with time, and the increase was represented as an approximately asymptotic growth curve (Fig 3). The median values at each quarter of the control group were found to be greater than those of the DS group, with the exception of y-pw at quarter 1.

The curve pattern of the DS group was also different from that of the healthy controls (Fig 3). The DS group showed an initial increase in width, depth, and volume of the hard palate but decreased with time to a local maximum (plateau). In controls, such local maximums are nonexistent in any parameter. The 1-dimensional parameters showed this plateau approximately after quarter 3, whereas the 3-dimensional volume had its maximum at quarter 2, followed by a slight depression, then increased again. Further noticeable discrepancies concerned palatal index (pi) and the distance y-pw, which represents the anterior part of the hard palate up to its maximum width. Y-pw showed a local minimum followed by a local maximum with a further decrease in the anterior dimension. The dimensionless palatal index differed between DS and controls.

## Discussion

The present study compares the morphometric findings of the hard palate between a group of infants with Down syndrome and a matched healthy control group. The following limitations of the study have to be taken into account when interpreting the results. Ethnicity has an influence on the morphology of the head and face. The DS group consisted of Caucasians only, therefore the results could not be extrapolated to other ethnic groups. DS individuals were taken from a patient group that was intended to treat with a stimulating plate [20]. This criteria may have biased the selection of a representative DS population. Moreover, measurements of the DS palates are not longitudinal data.

In the literature, a small maxilla in the anteroposterior or transverse dimension is often considered as maxillary hypoplasia [21]. The meaning of hypoplasia can differ, depending of the diagnostic tool used. Analyses of lateral head x-rays, CBCT images, plaster casts, ultrasound images, and MRI images may not compare with each other, but there is agreement in the literature that smaller dimensions are the consequence of maxillary underdevelopment [1].

Maxillary hypoplasia is of diagnostic interest in different stages of growth. During prenatal evaluation, the occurrence of maxillary hypoplasia may lead to the detection of structural abnormalities, genetic disorders, or syndromes [21]. During childhood, maxillary hypoplasia could be seen as a predictor of Angle Class III malocclusion [7, 9], obstructive sleep apnea [22], or both. After completion of growth, the palate of DS patients is often described as high arched and narrow [4, 11, 12, 14]. There is limited information whether maxillary hypoplasia causes permanent alteration of palatal morphology in later life.

We found that in the first year of life, palatal dimensions of the younger control group were consistently greater than those of the older DS group. No differences in measurements were observed after matching the groups by palatal volume, meaning that the hard palate of DS infants had a normal shape relative to width, length, and depth at this particular age. Fischer-Brandis [23] observed comparable dimensions in a 2–16-month-old DS group (Table 2) and rejected the hypothesis of high-arched, narrow palates.

**Table 2:** Studies measuring the hard palate of patients with Down syndrome.

Study	Cicero et al., 2004	Fischer-Brandis, 1985	Bhagyalakshmi et al., 2007	Skrinjarić et al., 2004	Abeleira et al., 2015	Westermann et al., 1974	Panchón-Ruiz et al., 2000	Dellavia et al., 2007
Age	11–14gw	2–16 m	6–16 y	10–20 y	10–40 y	16–29 y	20–29 y	20–45 y
DS male	88*	42	26	16	25	19	38	32
DS female		48	22	18	15	21	19	15
Control group (matched by)	839 (–)	–	48 (gender)	34 (age + gender)	40 (age + gender)	44 (–)	100 (–)	37 (–)
Palatal width (mm)	–	31.9 ± 2.6	35.3 ± 2.5	39.5 ± 2.9	30.2 ± 3.2	29.3	26.1 ± 3.7	36.5
Palatal depth (mm)	–	7.8 ± 1.1	16.3 ± 3.5	17.6 ± 2.5	12.8 ± 1.6	12.8	21.1 ± 2.0	12.4
Palatal index (depth/width)	–	0.2 ± 0.0	0.4 ± 1.4	0.4	0.4		0.4 ± 11.3	0.33
Palatal volume (mm^3^)	–	–	4270 ± 900	–	-	–	–	–
Palatal length (mm)	7.6	–	41.8 ± 3.4	38.6 ± 2.25	35.5 ± 2.9	–	23.1 ± 2.7	40

Data presented as Mean ± SD; [–] Indicates data not available;

^[^*^]^ Gender not given; gw–gestational weeks; m–months; d–days; y–years.

Further studies are available only for adolescent and adult DS patients. Bhagyalakshmi et al. [12] investigated 6–16-year-old DS children and found high-arched and narrow hard palates. Main differences existed in palatal depth and length, whereas palatal width and volume were similar to those in this study (Table 2). Dellavia et al. [11] found comparable dimensions in a group of 20–45-year-old DS patients (Table 2) that confirmed the results of Bhagyalakshmi et al. [12]. Panchón-Ruiz et al. [24] also found a characteristic palatal morphology in adult DS patients but measured a smaller palatal length and width than did other studies (Table 2). Abeleira et al. [25] investigated 10-40-year-old DS individuals and found narrow hard palates, whereas the length and the hight of the palates were comparable to an age matched control group.

Hypodontia is seen as a contributing factor in maxillary hypoplasia in DS patients [11], but it is unclear why it would affect predominantly width and depth rather than length of the palate. In our study, palatal length of DS subjects was the only steadily growing parameter (Fig 3; upper row, right). All other measurements approached a local extremum (Fig 3). This local maximum was present after approximately quarter 3, whereas the 3-dimensional volume had its maximum at quarter 2, followed by a slight depression, then increased again (Fig 3; lower row, right).

It was stated that palatal volume is a reliable indicator of growth [18]. If the palate is deformed by external factors [16, 17, 26] 1-dimensional measurements may be misleading in assessing growth of this 3-dimensional structure.

Only few studies have investigated the volume. Primožič et al. [19] observed a slight decrease in palatal volume during the transition to the early mixed dentition stage of healthy normals. The volume increased during the 30th month from 2948.9 ± 479.7 mm^3^ to 3306.6 ± 647.1 mm^3^ [19]. These values are below our own results in younger DS subjects. Bhagyalakshmi et al. [12] measured higher values, but age-related data were not presented.

It is apparent from the literature that high-arched and narrow palates are a characteristic feature in adult DS subjects. The DS palate develops differently in each plane of space in a wavelike overall growth pattern. DS infants may present maxillary hypoplasia but with normal shape of the palatal vault and alveolar ridges. It could be shown that growth patterns change at approximately quarter 3 at 6 to 9 month (≥183 days <274 days). Fischer-Brandis [23] observed normal shape in a DS group aged 60 to 487 days and Primožič et al. [19] detected a first decrease of palatal volume around 548 days at the end of the transition to the early mixed dentition.

Assuming that shape alterations start around these ages, it would be advantageous to begin oral muscular stimulating treatment between 6 and 18 months (183–548 days) of age. This is in accordance with recommendations to treat hypotonicity of the perioral and masticatory muscles, as well as lips, and a possible protruding tongue [15]. Treatment of oral muscular deficiency, tongue protrusion, and habitual mouth opening is ideal at the age of 17.9 months [15]. Significant improvement in lip closure and tongue position was found in a DS group in which treatment started at 6.5 months [27]. A narrow, disproportional palate is seen as a contributing factor for speech articulation disorders [12]. It has been shown speech development was faster and oral function improves better in a DS group treated at 4.8 months compared with a control group [28].

## Conclusions

The hard palate of infants with Down syndrome is of normal shape in the first 6 to 9 months of age but considerably smaller in all 3 dimensions compared with healthy normals. From the age of 6 to 9 months onward, the growth pattern of the hard palate varies in the various planes of space. Anatomical distortions such as high-arched, narrow shapes could therefore be interpreted as secondarily acquired in later life. To prevent palatal shape alterations and enhance oral function which also contributes to maxillary development it could be advantageous to begin oral muscular stimulating therapy between 6 and 9 months of age.

## Supporting information

S1 Table Raw data of the measurements obtained. (CSV)
