# Correction: Growth of the hard palate in infants with Down syndrome compared with healthy infants—A retrospective case control study

**DOI:** 10.1371/journal.pone.0203899

**Published:** 2018-09-07

**Authors:** Daniel Klingel, Ariane Hohoff, Robert Kwiecien, Dirk Wiechmann, Thomas Stamm

A reference is omitted from the second sentence of the third paragraph in the Introduction section.

The sentence should read: The hard palate of DS adolescents is often described as high arched and constricted or narrow [4, 11, 12], but data in the literature are contradictory [13, 14], depending on patient age [23] of the studied DS group.
